# Triglycerides and HDL cholesterol as strong correlates of insulin resistance: Evidence from NHANES 2013 to 2018

**DOI:** 10.1097/MD.0000000000048542

**Published:** 2026-05-08

**Authors:** Zhenze Yu, Zihan Zhao, Ming Zhuang Sun, Yalei Han

**Affiliations:** a Cardiac Department, Aerospace Center Hospital, Beijing, China; b Cardiac Department, Peking University Aerospace School of Clinical Medicine, Beijing, China; c Interventional Center of Valvular Heart Disease, Beijing Anzhen Hospital, Capital Medical University, Beijing, China.

**Keywords:** HDLC, insulin resistance, LDLC, metabolic syndrome, nutrition surveys, triglycerides

## Abstract

Insulin resistance (IR) is a key factor in metabolic disorders. While lipid abnormalities often coexist with IR, their independent associations remain unclear. We analyzed 4698 adults from National Health and Nutrition Examination Survey (2013–2018). The Homeostasis Model Assessment (HOMA) of IR was calculated from fasting glucose and insulin. Lipid profiles (LDLC, HDLC, total cholesterol, triglycerides [TG]) were examined in relation to HOMA-IR using multivariable-adjusted regression models. HDLC showed a inverse association with HOMA-IR (*β* = −0.877, *P* < .001), while TG had a correlation (*β* = 0.679, *P* < .001). LDLC and total cholesterol exhibited weaker, nonlinear relationships. High TG (Q4 odds ratio = 5.53) and low HDLC (Q4 odds ratio = 0.15) were predictive of elevated HOMA-IR (*P* < .001). TG and HDLC are associated with HOMA-IR, with distinct dose-response patterns. These findings emphasize the importance of lipid management in metabolic health.

## 1. Introduction

Insulin resistance (IR) is a fundamental pathophysiological mechanism underlying metabolic disorders, including type 2 diabetes mellitus, dyslipidemia, and cardiovascular disease (CVD).^[1]^ The Homeostasis Model Assessment (HOMA) of IR is a widely used surrogate marker for IR, calculated from fasting insulin and fasting blood glucose (FBG) levels.^[2]^ While the hyperinsulinemic-euglycemic clamp (HEC) remains the gold standard for assessing insulin sensitivity, HOMA-IR offers a practical alternative for large-scale epidemiological studies due to its simplicity and correlation with HEC-derived measures.^[3]^

Growing evidence indicates that HOMA-IR is closely associated with both glucose metabolism dysfunction and dyslipidemia. IR often coexists with lipid abnormalities (such as hypertriglyceridemia and low high-density lipoprotein cholesterol (HDLC)) while further exacerbating metabolic disturbances, forming a vicious cycle that elevates the risk of CVD and type 2 diabetes mellitus.^[4–7]^

Additionally, while low-density lipoprotein cholesterol (LDLC) and total cholesterol (TC) are well-established CVD risk factors, their independent associations with IR remain controversial.This may be due to alterations in LDL metabolic kinetics under insulin-resistant conditions, as well as the confounding interference of lipid-lowering agents (such as statins) widely used in clinical studies on lipid profiles.

This study leverages the National Health and Nutrition Examination Survey (NHANES) data (2013–2018) to explore the associations between lipid profiles (LDLC, HDLC, TC, and triglycerides (TG)) and HOMA-IR, while adjusting for potential confounders.

## 2. Methods

### 2.1. Study population

This study utilized data from participants in NHANES cycles spanning 2013 to 2014, 2015 to 2016, and 2017 to 2018. The initial pooled sample comprised 29,400 individuals.

**Figure 1.**
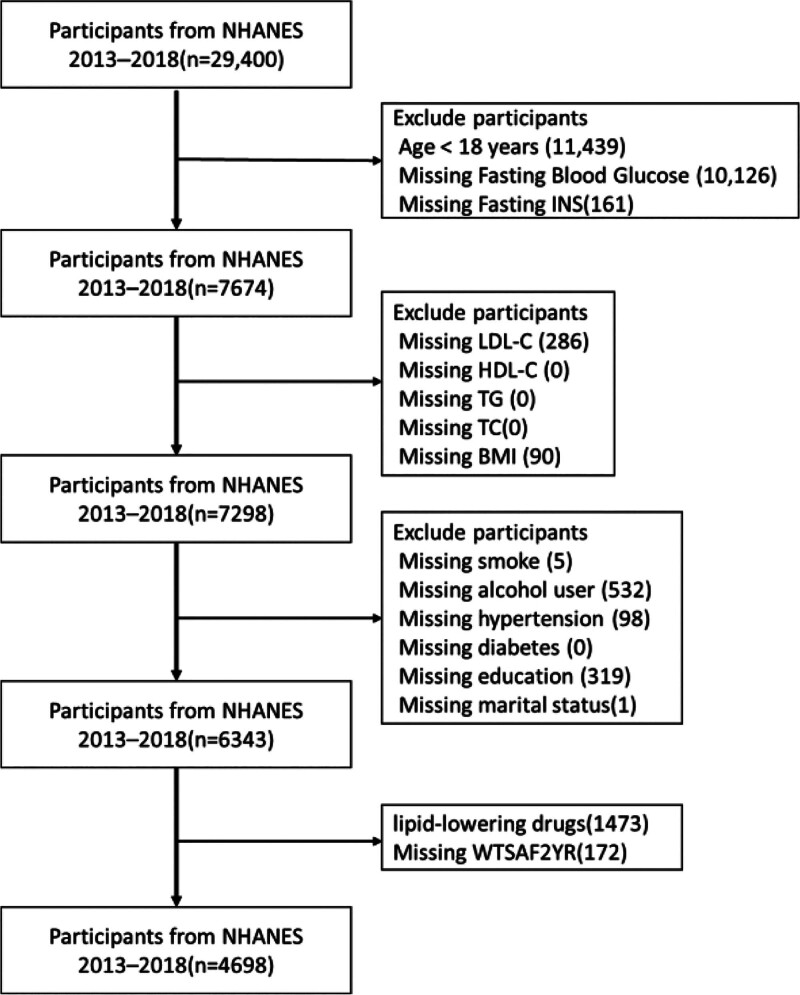
Patient selection flow diagram. Selection process of participants from the NHANES 2013 to 2018 cycles. Initial sample of 29,400 individuals was sequentially excluded based on age, missing data for key variables (fasting glucose, insulin, lipid profiles, BMI, covariates), use of lipid-lowering medication, and missing sampling weights, resulting in a final analytic sample of 4698 participants. BMI = body mass index, HDLC = high-density lipoprotein cholesterol, INS = insulin, LDLC = low-density lipoprotein cholesterol, MEC = mobile exam center, n = number of participants, NHANES = National Health and Nutrition Examination Survey, TC = total cholesterol, TG = triglycerides, WTSAF2YR = fasting subsample 2 year.

From 29,400 NHANES 2013 to 2018 participants, we excluded those aged < 18 years (n = 11,439), missing FBG (n = 10,126), missing insulin (n = 161), missing LDLC (n = 286), missing body mass index (BMI) (n = 90), missing covariates (smoking/alcohol/hypertension/education/marital status, n = 955), using lipid-lowering drugs (Table S1) (n = 1473), or missing sampling weights (n = 172). A total of 4698 participants were included (Fig. 1)

### 2.2. HOMA-IR and lipid parameters

HOMA-IR was calculated as: HOMA-IR = (fasting insulin (μU/mL) × FBG (mmol/L))/22.5. Serum levels of LDLC (mmol/L), HDLC (mmol/L), TC (mmol/L), and TG (mmol/L) were measured in blood samples obtained after a minimum 8-hour overnight fast.

## 3. Statistical analysis

Continuous variables are expressed as median (interquartile range) and categorical variables as n (%). Differences across HOMA-IR tertiles were assessed using Kruskal-Wallis tests (continuous) and chi-square tests (categorical). HOMA-IR variations across lipid Tertiles (LDLC/HDLC/TC/TG) were analyzed via Analysis of Covariance, adjusted for age, sex, race, education, marital status, smoking, alcohol, BMI, hypertension, and diabetes. Linear associations used the same adjustment model in multiple regression. Logistic regression employed hierarchical models: Model 1 (age + sex), Model 2 (Model 1 + sociodemographics + lifestyle + BMI), and Model 3 (Model 2 + hypertension + diabetes). Restricted cubic splines assessed nonlinearity. All analyses, including Analysis of variance, linear regression, logistic regression, and restricted cubic splines, accounted for the complex survey design of NHANES by incorporating appropriate sampling weights. All statistics were completed using R version 4.3.1 (Lucent Technologies, Murray Hill).

## 4. Results

### 4.1. Characteristics of the study population

A total of 4698 participants were included in the final analysis. Participants in the higher HOMA-IR tertiles (T2 and T3) were older, with median ages of 45 and 47 years compared to 42 years in T1 (*P* < .001). Sex distribution was similar across tertiles (*P* = .81), but racial composition varied with a higher proportion of Mexican Americans in T3 (21.6%) compared to T1 (9.8%) (*P* < .001).Education levels also differed, with fewer individuals in T3 having college or higher education (52.1%) compared to T1 (61.6%) (*P* < .001). Metabolic markers such as LDLC, HDLC, TC, TG, and BMI exhibited trends across tertiles (all *P* < .001), with participants in higher HOMA-IR tertiles had higher median LDLC, higher TC, higher TG and lower HDLC. Notably, hypertension and diabetes prevalence increased markedly in T3 (45.8% and 27.7%, respectively) compared to T1 (26.3% and 4.9%) (*P* < .001). Smoking rates were similar across groups (*P* = .39), while alcohol consumption was slightly lower in T3 (75.4%) compared to T1 (79.1%) (*P* = .036) (Table 1).

**Table 1:** Baseline characteristics of the all participant by HOMA-IR tertiles.

	Overall	T1	T2	T3	*P* value
N	4698	1566	1566	1566	
HOMA-IR (median [IQR])	2.25 [1.37, 3.81]	1.11 [0.83, 1.37]	2.25 [1.93, 2.66]	4.93 [3.81, 7.20]	< .001
Age (median [IQR])	45.00 [32.00, 59.00]	42.00 [30.00, 58.00]	45.00 [32.00, 60.00]	47.00 [34.00, 60.00]	< .001
Sex (male %)	2216 (47.2)	729 (46.6)	747 (47.7)	740 (47.3)	.81
Race (%)					
Mexican American	759 (16.2)	154 (9.8)	266 (17.0)	339 (21.6)	< .001
Other Hispanic	532 (11.3)	159 (10.2)	171 (10.9)	202 (12.9)	
Non-Hispanic White	1687 (35.9)	654 (41.8)	534 (34.1)	499 (31.9)	
Non-Hispanic Black	961 (20.5)	299 (19.1)	341 (21.8)	321 (20.5)	
Other Race - Including Multiracial	759 (16.2)	300 (19.2)	254 (16.2)	205 (13.1)	
Education (%)					
Middle school or lower	958 (20.4)	251 (16.0)	333 (21.3)	374 (23.9)	< .001
High school	1057 (22.5)	351 (22.4)	330 (21.1)	376 (24.0)	
College or more	2683 (57.1)	964 (61.6)	903 (57.7)	816 (52.1)	
Marital (%)					
Married	2337 (49.7)	724 (46.2)	824 (52.6)	789 (50.4)	.005
Divorce	492 (10.5)	165 (10.5)	154 (9.8)	173 (11.0)	
Other	1869 (39.8)	677 (43.2)	588 (37.5)	604 (38.6)	
FPG (mmol/L) (median [IQR])	5.55 [5.22, 6.05]	5.27 [4.94, 5.55]	5.55 [5.27, 5.94]	6.00 [5.55, 6.77]	< .001
LDLC (mmol/L)(median [IQR])	2.95 [2.38, 3.57]	2.77 [2.22, 3.39]	3.00 [2.46, 3.62]	3.05 [2.51, 3.62]	< .001
HDLC (mmol/L) (median [IQR])	1.34 [1.11, 1.63]	1.55 [1.29, 1.86]	1.34 [1.14, 1.60]	1.19 [1.01, 1.40]	< .001
TC (mmol/L)(median [IQR])	4.91 [4.27, 5.59]	4.76 [4.16, 5.48]	4.97 [4.29, 5.63]	4.97 [4.34, 5.64]	< .001
TG (mmol/L) (median [IQR])	1.02 [0.69, 1.50]	0.76 [0.54, 1.11]	1.00 [0.69, 1.45]	1.33 [0.96, 1.92]	< .001
BMI (kg/m^2^) (median [IQR])	27.90 [24.10, 32.90]	23.90 [21.50, 26.90]	27.80 [24.83, 31.60]	33.10 [28.80, 38.20]	< .001
Smoke (%)	1943 (41.4)	669 (42.7)	633 (40.4)	641 (40.9)	.39
Alcohol (%)	3644 (77.6)	1239 (79.1)	1224 (78.2)	1181 (75.4)	.036
Hypertension (%)	1666 (35.5)	412 (26.3)	536 (34.2)	718 (45.8)	< .001
Diabetes (%)	655 (13.9)	77 (4.9)	144 (9.2)	434 (27.7)	< .001

Data are expressed as the median (upper and lower quartiles) or number (%).

BMI = body mass index, FPG = fasting plasma glucose, HDLC = high-density lipoprotein cholesterol, HOMA-IR = Homeostatic Model Assessment of Insulin Resistance, IQR = interquartile range, LDLC = low-density lipoprotein cholesterol, TC = total cholesterol, TG = triglycerides.

Bold indicates *P* value < .05.

### 4.2. Association between lipid profiles and HOMA-IR

Table 2 demonstrates variations in HOMA-IR levels across tertiles of different lipid parameters after adjusting for multiple covariates.

**Table 2:** HOMA-IR levels across the Tertiles of LDLC, HDLC, TG and TC.

	Tertiles 1	Tertiles 2	Tertiles 3	*P* value
LDLC	2.132 (1.817,2.448)	2.306 (2.194,2.698)***	2.300 (1.891,2.710)**	< .001
HDLC	1.052 (0.877,1.227)	0.912 (0.694,1.129)***	0.783 (0.572,0.993)***,###	< .001
TC	3.915 (3.568,4.261)	4.071 (3.629,4.513)**	4.067 (3.605,4.528)*	.006
TG	1.599 (1.297,1.902)	1.867 (1.505,2.229)***	2.112 (1.770,2.533)***,###	< .001

Data are expressed as adjusted mean and 95% CI.

BMI = body mass index, CI = confidence interval, HDLC = high-density lipoprotein cholesterol, HOMA-IR = Homeostatic Model Assessment of Insulin Resistance, LDLC = low-density lipoprotein cholesterol, TC = total cholesterol, TG = triglycerides.

P values were performed by analysis of covariance (ANCOVA) adjusted for age, sex, race, education, marital, smoke, alcohol, BMI, hypertension and diabetes.

Symbols indicate statistical significance compared to the reference group.

* .

# *P* < .05.

** .

## *P* < .01.

*** .

## .

# *P* < .001.

* Compared with Tertiles 1.

# Compared with Tertiles 2.

For LDLC, HOMA-IR showed a increase from Tertiles 1 (2.132, 95% confidence interval [CI]: 1.817–2.448) to Tertiles 2 (2.306, 95% CI: 2.194–2.698, *P* < .001) and Tertiles 3 (2.300, 95% CI: 1.891–2.710, *P* < .01), a association with HOMA-IR(*P* for trend < .001). HDLC displayed an inverse relationship, with HOMA-IR levels decreasing from Tertiles 1 (1.052, 95% CI: 0.877–1.227) to Tertiles 3 (0.783, 95% CI: 0.572–0.993, *P* < .001).

**Figure 2.**
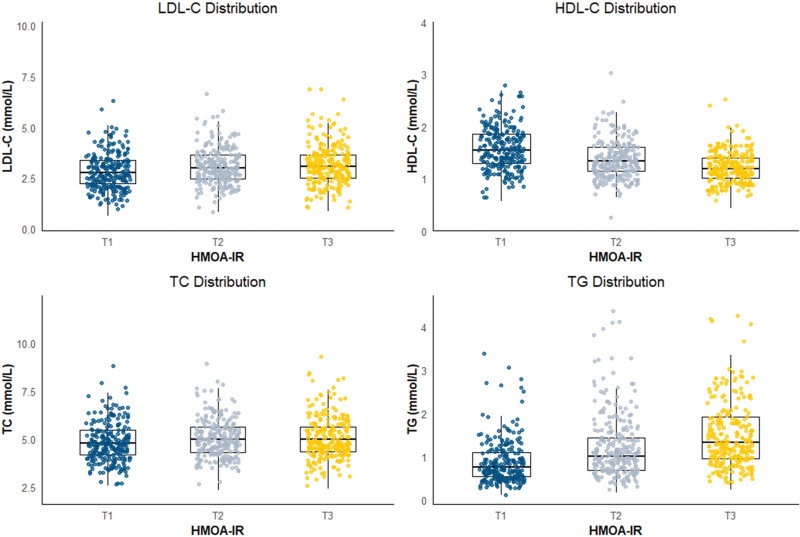
Distributions of LDLC, HDLC, TC, and TG levels across HOMA-IR tertiles. Estimated marginal means (95% CI) of HOMA-IR according to tertiles of LDLC, HDLC, TC, and TG. Significant trends were observed for all lipids, with the most pronounced associations seen for HDLC and TG. CI = confidence interval, HDLC = high-density lipoprotein cholesterol, HOMA-IR = Homeostasis Model Assessment, LDL = low-density lipoprotei, TC = total cholesterol, TG = triglycerides.

TC showed a modest but statistically association, with HOMA-IR increasing from Tertiles 1 (3.915, 95% CI: 3.568–4.261) to higher tertiles (Tertiles 2: 4.071, *P* < .01; Tertiles 3: 4.067, *P* < .05). The most pronounced effect was observed for TG, where HOMA-IR levels rose sharply from Tertiles 1 (1.599, 95% CI: 1.297–1.902) to Tertiles 3 (2.112, 95% CI: 1.770–2.533, *P* < .001), a correlation with HOMA-IR (Fig. 2).

Linear regression analysis revealed distinct associations between lipid parameters and HOMA-IR after full adjustment. HDLC showed a inverse relationship with HOMA-IR (*β* = −0.877, 95% CI: −1.127 –−0.627; *P* < .001), while TG demonstrated a correlation (*β* = 0.679, 95% CI: 0.531–0.827; *P* < .001). In contrast, neither LDLC (*β* = 0.026, *P* = .818) nor TC (*β* = 0.045, *P* = .673) showed associations with HOMA-IR (Table 3).

**Table 3:** Linear regression analysis for the correlation of LDLC, HDLC, TC, TG and HOMA-IR in all participants.

	HOMA-IR	
	*β* (95% CI)	*P* value
LDLC	0.026 (−0.202, 0.254)	.818
HDLC	−0.877 (−1.127, −0.627)	< .001
TC	0.045 (−0.171, 0.262)	.673
TG	0.679 (0.531, 0.827)	< .001

Data are expressed as standardized coefffcients (*β*) and 95% CI.

BMI = body mass index, CI = confidence interval, HDLC = high-density lipoprotein cholesterol, HOMA-IR = Homeostatic Model Assessment of Insulin Resistance, LDLC = low-density lipoprotein cholesterol, TC = total cholesterol, TG = triglycerides.

*P* values were adjusted for age, sex, race, education, marital, smoke, alcohol, BMI, hypertension and diabetes.

### 4.3. Lipid profiles and HOMA-IR risk

Multivariable analysis showed HDLC maintained inverse associations with HOMA-IR (Q4 odds ratio [OR] = 0.15, *P* < .001), while TG demonstrated the relationship (Q4 OR = 5.53, *P* < .001). LDLC and TC associations attenuated after full adjustment (LDLC Q4 OR = 1.34, *P* = .11; TC Q4 OR = 1.42, *P* < .05). After full adjustment for demographics, BMI, and comorbidities, the ORs for elevated HOMA-IR in the highest quartile relative to the lowest quartile were 0.15 for HDLC (95% CI: 0.11–0.22, *P* < .001), 5.53 for TG (95% CI: 3.89–7.85, *P* < .001), 1.34 for LDLC (95% CI: 1.05–1.71, *P* < .05), and 1.42 for TC (95% CI: 1.08–1.88, *P* < .05) (Table 4).

**Table 4:** Logistic regression analysis for the association of LDL-C, HDLC, TC, TG and HOMA-IR.

	OR (95% CI)				*P* for trend
	Quartile 1	Quartile 2	Quartile 3	Quartile 4	
LDLC					
Model 1	1.00 (Ref)	1.49 (1.17, 1.90)***	1.67 (1.24, 2.24)***	1.63 (1.27, 2.09)***	< .001
Model 2	1.00 (Ref)	1.17 (0.89, 1.54)	1.03 (0.74, 1.42)	1.25 (0.98, 1.59)	.2
Model 3	1.00 (Ref)	1.19 (0.90, 1.58)	1.13 (0.82, 1.56)	1.34 (1.05, 1.71)*	.11
HDL-C					
Model 1	1.00 (Ref)	0.50 (0.40, 0.62)***	0.26 (0.20, 0.32)***	0.07 (0.05, 0.09)***	< .001
Model 2	1.00 (Ref)	0.61 (0.46, 0.81)***	0.38 (0.28, 0.52)***	0.14 (0.10, 0.20)***	< .001
Model 3	1.00 (Ref)	0.63 (0.47, 0.83)***	0.40 (0.29, 0.55)***	0.15 (0.11, 0.22)***	< .001
TC					
Model 1	1.00 (Ref)	1.20 (0.95, 1.51)	1.12 (0.85, 1.47)	1.29 (0.99, 1.69)	.2
Model 2	1.00 (Ref)	1.01 (0.75, 1.36)	0.95 (0.72, 1.25)	1.30 (0.98, 1.72)	.2
Model 3	1.00 (Ref)	1.10 (0.82, 1.49)	1.03 (0.78, 1.35)	1.42 (1.08, 1.88)*	.068
TG					
Model 1	1.00 (Ref)	2.42 (1.79, 3.27)***	4.24 (3.26, 5.52)***	8.24 (6.17, 11.0)***	< .001
Model 2	1.00 (Ref)	2.07 (1.36, 3.15)***	3.32 (2.43, 4.55)***	5.99 (4.29, 8.36)***	< .001
Model 3	1.00 (Ref)	1.99 (1.32, 3.01)***	3.18 (2.30, 4.39)***	5.53 (3.89, 7.85)***	< .001

Data are expressed as adjusted mean and 95% CI.

Model 1: age,sex.

Model 2: Model 1 + race, education, marital, smoke, alcohol, BMI.

Model 3: Model 2 + hypertension and diabetes.

BMI = body mass index, CI = confidence interval, HDLC = high-density lipoprotein cholesterol, HOMA-IR = Homeostatic Model Assessment of Insulin Resistance, LDLC = low-density lipoprotein cholesterol, OR = odds ratio, TC = total cholesterol, TG = triglycerides.

* *P* < .05.

** *P* < .01.

*** *P* < .001.

* Compared with Quartile 1.

### 4.4. Nonlinear associations between lipids and HOMA-IR

**Figure 3.**
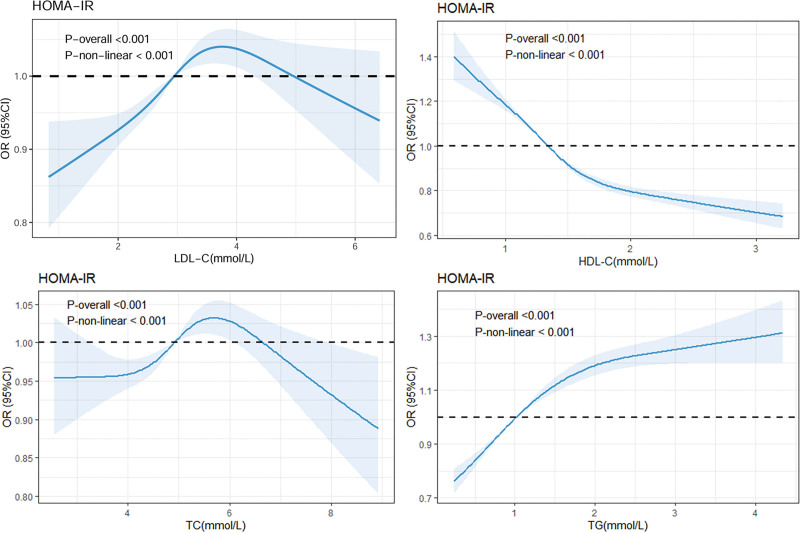
Nonlinear associations of HOMA-IR with LDLC, HDLC, TC, and TG levels. Restricted cubic spline plots depicting the adjusted association between each lipid parameter (LDLC, HDLC, TC, TG) and HOMA-IR. Nonlinear relationships were identified for all lipids (*P* for nonlinearity < .05). CI = confidence interval, HDLC = high-density lipoprotein cholesterol, HOMA-IR = Homeostasis Model Assessment, LDL = low-density lipoprotein, OR = odds ratio, TC = total cholesterol, TG = triglycerides.

Nonlinear analysis revealed nonlinear relationships between lipid parameters and HOMA-IR. HDLC demonstrated a inverse association with HOMA-IR. Conversely, TG exhibited a pronounced relationship, with risk escalating exponentially beyond threshold levels. LDLC also showed nonlinear associations (*P* < .001), complex dose-response patterns (Fig. 3).

## 5. Discussion

Our study confirmed the independent associations of specific lipid fractions with HOMA-IR after comprehensive confounder adjustment, and further characterized their nonlinear dose-response relationships in a general United States (US) adult population.These findings align with previous research indicating that hypertriglyceridemia and low HDLC are hallmarks of IR and metabolic syndrome.^[8–10]^

The strengths of associations varied across adjusted models (Table 4). HDLC and TG showed the most robust associations, remaining highly significant across all models (HDLC Q4 OR = 0.15, TG Q4 OR = 5.53 in Model 3, both *P* < .001), indicating independence from demographic, lifestyle, and metabolic confounders. In contrast, LDLC and TC were significantly associated with HOMA-IR in the minimally adjusted model (Model 1) but attenuated substantially after adjusting for BMI and metabolic comorbidities (Models 2 and 3), suggesting their apparent associations are largely explained by confounding or mediation by obesity and related conditions rather than independent effects.

A study of 614 men and 1055 women (nondiabetic) demonstrated significant associations between IR and TG/HDLC ratio, TC/HDLC ratio, and TG levels.^[11]^A study of 75 familial mixed hyperlipidemia patients and 75 healthy controls revealed significantly elevated HOMA-IR in familial mixed hyperlipidemia subjects, accompanied by an atherogenic lipid profile characterized by hypertriglyceridemia, low HDLC, elevated small dense LDL, and increased apolipoprotein B levels.^[12]^

A study of 544 subjects demonstrated that dyslipidemia (high TG, low HDLC) in normal glucose tolerance individuals was significantly associated with elevated HOMA-IR and reduced β-cell function. In impaired glucose regulation patients, dyslipidemia further exacerbated β-cell dysfunction, particularly in combined glucose intolerance subgroups. Multivariate regression confirmed correlations between TG/HDLC and HOMA-IR, while elevated TG and TC negatively correlated with β-cell compensatory capacity.^[13]^

A genome-wide association study identified many genetic loci that link TG/HDL with IR.^[14]^ Some studies have found that the IR markers in the liver and adipose tissue are respectively closely related to elevated TG and decreased HDL.^[15]^ Additionally, some glycerophospholipids have also been found to be closely related to IR.^[16]^ In these studies, TG, the ratio of TG to HDLC, and IR are closely related. They have shown excellent diagnostic value in identifying metabolic syndrome and IR.^[9,17–19]^ More importantly, this simple ratio is not only a marker of IR, but also has a significant association with hard outcomes (including carotid intima-media thickness and long-term cardiovascular events).^[20]^

The relationship between LDLC levels and IR remains controversial. A study of 26,746 healthy US women demonstrated that participants with HOMA-IR > 1.34 (median) showed significantly higher TC and LDLC levels compared to those with lower HOMA-IR.^[21]^ A study in Hong Kong involving 2649 subjects showed that LDLC and TC levels increased significantly with the increase of HOMA-IR.^[22]^

However, there are also some opposite viewpoints. A study that included 378 male subjects and 509 female subjects showed that multiple regression analysis revealed that HDLC was independently associated with HOMA-IR, and LDLC was associated with HOMA-IR, but after adjusting for confounding factors, there was no significant association.^[23]^A study from the Czech Republic showed that among 1840 participants, HOMA-IR increased with the increase of LDLC concentration, but the difference in the average value was not statistically significant.^[24]^ A fundamental study demonstrated that IR was correlated with capillary isotachophoresis fast-migrating (f) LDL levels (*P* < .01). The relationship between HOMA-IR and capillary isotachophoresis fast-migrating LDL levels was dependent on TG levels but independent of BMI and LDL particle size.^[25]^

There is a complex bidirectional relationship between glucose and lipid metabolism. Diabetic dyslipidemia is not only a consequence of IR but may also contribute to glucose metabolism disorders by affecting IR,^[26]^ Although statin therapy improves lipid profiles, it may modestly increase the risk of new-onset diabetes (elevated HOMA-IR) due to inhibition of β-hydroxy-β-methylglutaryl-coenzyme A reductase.^[27–30]^ Inhibition of β-hydroxy-β-methylglutaryl-coenzyme A reductase by statins not only reduces cholesterol synthesis but may also impair insulin signaling and glucose uptake in peripheral tissues, leading to reduced insulin sensitivity.^[27–30]^The relationship between lipoprotein(a) and diabetes risk remains inconclusive.^[21,31,32]^ This further highlights the differential effects of distinct lipoprotein components on IR.

## 6. Limitations

This study has several limitations. First, its cross-sectional design prevents causal inference. Second, HOMA-IR, while practical, is less precise than gold-standard methods like the HEC. Third, excluding lipid-lowering medication users limits generalizability to treated populations. The excluded patients usually have a higher cardiovascular risk, a longer history of metabolic abnormalities, and more severe lipid disorders. Even when receiving treatment, they may still not reach the ideal metabolic control targets. Therefore, the conclusion of this study is mainly applicable to the general population who have not received lipid-lowering treatment, and cannot be directly extended to patients who have already received treatment. Fourth the US-based NHANES sample may not fully represent other ethnic or geographic groups.

Fifth, the potential impacts of multicollinearity and overfitting. The lipid parameters (LDLC, HDLC, TC, TG) included in this study have intrinsic biological correlations. TC is the sum of LDLC, HDLC, and other lipoprotein cholesterol. Moreover, these lipid indicators are highly correlated with metabolic variables such as BMI, blood sugar, and hypertension. This complex interrelationship may introduce multicollinearity, resulting in an increase in the standard error of effect estimates in the regression model.

Finally, this study did not include major cardiovascular events as outcomes, as the cross-sectional nature of NHANES and its reliance on self-reported cardiovascular history may introduce recall bias and limit causal inference. Future prospective studies with adjudicated cardiovascular endpoints are warranted to validate and extend our findings. Future longitudinal or mechanistic studies could clarify these associations.

Although the present study confirms that TG and HDLC are core lipid markers of IR in untreated populations, caution is warranted when extrapolating these findings to the clinically common population of patients who have not achieved lipid treatment goals. Future research specifically targeting this population is needed to inform more precise lipid management strategies.

## 7. Conclusion

TG and HDLC are strongly associated with HOMA-IR, with distinct dose-response patterns. These findings emphasize the importance of lipid management in metabolic health.

## Author contributions

**Conceptualization:** Yalei Han.

**Data curation:** Zhenze Yu.

**Formal analysis:** Zhenze Yu.

**Investigation:** Ming Zhuang Sun.

**Resources:** Ming Zhuang Sun.

**Software:** Zihan Zhao.

**Supervision:** Yalei Han.

**Writing – original draft:** Zhenze Yu.

**Writing – review & editing:** Yalei Han.


