# Supplementary material for: Triglycerides and HDL cholesterol as strong correlates of insulin resistance: Evidence from NHANES 2013 to 2018
Source: Medicine (Baltimore). 2026 May 8;105(19):e48542. doi: 10.1097/MD.0000000000048542 (PMC13166817; doi:10.1097/MD.0000000000048542)
Supplement: Supplementary file 1 [file medi-105-e48542-s001.docx]

Supplement 1. Lipid-Lowering Drugs Included in the NHANES Study Analysis.

| **Lipid-lowering drugs** | **Drug ID** |
| --- | --- |
| AMLODIPINE; ATORVASTATIN | d05048 |
| ANTIHYPERLIPIDEMIC AGENTS – UNSPECIFIED | c00019 |
| ATORVASTATIN | d04105 |
| CHOLESTYRAMINE | d00193 |
| COLESEVELAM | d04695 |
| COLESTIPOL | d00744 |
| EVOLOCUMAB | d08383 |
| EZETIMIBE | d04824 |
| EZETIMIBE; SIMVASTATIN | d05348 |
| FENOFIBRATE | d04286 |
| FENOFIBRIC ACID | d07371 |
| FLUVASTATIN | d03183 |
| GEMFIBROZIL | d00245 |
| HMG-COA REDUCTASE INHIBITORS – UNSPECIFIED | c00173 |
| ICOSAPENT | d07891 |
| LOVASTATIN | d00280 |
| NIACIN | d00314 |
| OMEGA-3 POLYUNSATURATED FATTY ACIDS | d00497 |
| PITAVASTATIN | d07637 |
| PRAVASTATIN | d00348 |
| ROSUVASTATIN | d04851 |
| SIMVASTATIN | d00746 |
| SIMVASTATIN; SITAGLIPTIN | d07805 |

Supplement 1. This table lists all lipid-lowering drugs included in the analysis of the National Health and Nutrition Examination Survey (NHANES) data. Each row represents a single drug or a fixed-dose combination product. The "Lipid-lowering drugs" column indicates the generic name(s) or combination name, and the "Drug ID" column provides the unique identifier code used in the NHANES database for each drug.
